# Prognostic significance of right ventricular dysfunction in heart failure with preserved ejection fraction: a meta-analysis of reconstructed time-to-event data

**DOI:** 10.1186/s44156-025-00080-5

**Published:** 2025-05-29

**Authors:** Roozbeh Narimani-Javid, Mehrdad Mahalleh, Kiyarash Behboodi, Kasra Izadpanahi, Alireza Arzhangzadeh, Reza Nikfar, Seyed Ali Hosseini, Ehsan Amini-Salehi, Sasan Shafiei, Hamed Vahidi, Kaveh Hosseini, Hamidreza Soleimani

**Affiliations:** 1 Research Center for Advanced Technologies in Cardiovascular Medicine, Cardiovascular Diseases Research Institute, Tehran University of Medical Sciences, Tehran, Iran; 2 Cardiovascular Diseases Research Institute, Tehran Heart Center, Tehran University of Medical Sciences, Tehran, Iran; 3 School of Medicine, Tehran University of Medical Sciences, Tehran, Iran; 4 Department of Cardiology, Shiraz University of Medical Sciences, Shiraz, Iran; 5 School of Medicine, Babol University of Medical Sciences, Mazandaran, Iran; 6 Department of Internal Medicine, Guilan University of Medical Sciences, Rasht, Iran; 7 Department of Cardiology, Imam Khomeini Hospital Complex, Tehran University of Medical Sciences, Tehran, Iran

**Keywords:** Heart failure with preserved ejection fraction, Diastolic heart failure, Right ventricular dysfunction, Time to event

## Abstract

**Background:**

The prognosis of Heart failure with preserved ejection fraction (HFpEF) is significantly impacted by the existence and severity of comorbidities. Recent studies highlight the right ventricle (RV) as a crucial player in heart failure pathophysiology. However, there are still gaps in understanding how right ventricular dysfunction (RVD) affects long-term outcomes in patients with heart failure with preserved ejection fraction (HFpEF).

**Materials and methods:**

In this systematic review and meta-analysis, a comprehensive search was conducted to identify studies investigating RVD as the predictor of the composite outcome of All-cause death, cardiac death, and hospitalization for HF in patients with HFpEF published until October 2024. RVD was defined as the deviation of at least one measurement of RV function from the recommended normal range based on modality and the normal ranges established in each study. Time and survival probability were extracted for each Group (HFpEF patients with and without RVD) in each of the Kaplan-Meier curves. Individual patient data were reconstructed by processing the extracted time points, survival probabilities, and the number of patients at risk in a two-stage approach. The restricted mean survival time (RMST) was also calculated as the area under the survival curve for each group.

**Results:**

Seven studies met the inclusion criteria, comprising 1936 individuals, of which 555 patients had RVD. The pooled prevalence of RVD among HFpEF was 41.2% (95% CI: 36.5; 45.9). Patients with RVD had a significantly higher risk of adverse outcomes compared to those without RVD, with an HR of 2.28 (95% CI, 1.95; 2.68, *p*-value < 0.001) in the eight-year follow-up after the RVD diagnosis. The one-year landmark analysis revealed that the majority of the event-free survival disparity between patients with RVD and those without arises from the first year after an RVD diagnosis. Patients with RVD also had shorter event-free survival. (ΔRMST = -2.127 years, 95% CI, -2.383; -1.872, *p*-value < 0.001).

**Conclusion:**

The development of RVD in HFpEF is linked to significantly increased composite outcomes of all-cause death and HF hospitalization and shorter event-free survival.

**Supplementary Information:**

The online version contains supplementary material available at 10.1186/s44156-025-00080-5.

## Graphical abstract

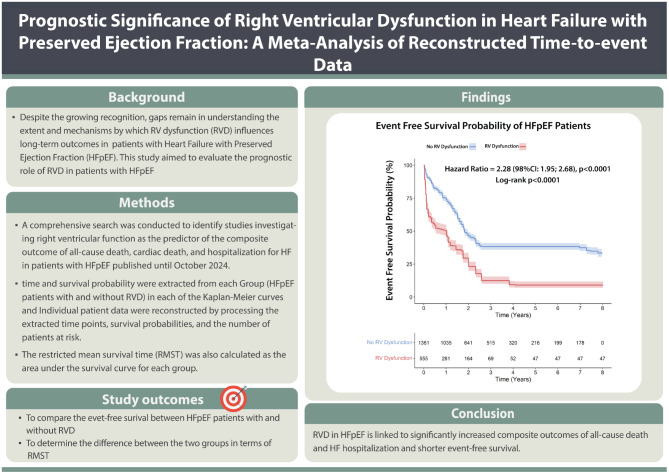

## Introduction

Heart failure with preserved ejection fraction (HFpEF) constitutes nearly half of all heart failure cases [1]. It is becoming recognized as a significant worldwide health concern, with an estimated five-year death rate of 75% [2–4]. The prognosis of patients with HFpEF is substantially impacted by the existence and severity of common comorbidities, including diabetes, atrial fibrillation, hypertension, coronary artery disease, peripheral artery disease, pulmonary hypertension, and chronic renal disease [5–9].

The prognostic significance of right ventricle dysfunction (RVD) in heart failure has long been undervalued and has only lately been recognized as an independent predictor of morbidity and mortality in these patients [10, 11]. Although the prognostic effects of RVD in patients with HFpEF have been demonstrated in previous meta-analyses [12, 13], interpreting the hazard ratio (HR) as an effect measure has limitations in clinical settings.

A valid HR analysis relies on the proportional hazards assumption, meaning that the hazard curves ratio must remain constant over time. In practice, this assumption often proves unrealistic, and if the proportional hazards assumption is violated, the HR may lack sufficient statistical power to identify a genuine exposure effect [14, 15]. Moreover, since HRs evaluate relative rather than absolute effects, it can be challenging to communicate this information to patients and trialists, particularly concerning the influence of the exposure and what patients can reasonably expect as outcomes from an exposure over time [16, 17].

Restricted mean survival time (RMST) is derived from the area under the survival curve. It is a model-free metric that does not rely on hazard assumptions and represents the average time without events until a specified point. Delta (Δ) RMST serves as an alternative metric for evaluating treatment benefits and determining statistical significance. This measure provides a clear absolute survival-time difference related to a treatment or risk factor, making it more understandable for both clinicians and patients [18].

This study aimed to assess ΔRMST alongside conventional HRs by reconstructing individual patient data from Kaplan-Meier curves, comparing the risk of adverse outcomes and event-free survival between HFpEF patients with RVD and those without RVD to better address the aforementioned limitations.

## Methods

### Study design

This systematic review and meta-analysis study was designed and conducted in accordance with the Preferred Reporting System for Systematic Reviews and Meta-Analysis (PRISMA) 2020 [19]. The study protocol was registered in the International Prospective Register of Systematic Reviews (PROSPERO) under the registration number CRD42024600369. Ethical approval was not required as no human or animal subjects were involved in this research.

### Search strategy

A comprehensive search was conducted to identify studies investigating right ventricular function as the predictor of adverse outcomes in HFpEF published until October 2024. We searched three electronic databases -PubMed, Scopus, and EMBASE- using a search syntax based on two key terms: “right ventricular function” AND “heart failure with preserved ejection fraction.” The detailed search syntax for each database is provided in Table S1.

### Eligibility criteria

Original observational studies were considered as included if they met the following criteria: (1) the study included patients diagnosed with HFpEF, based on the guidelines available at the time of the study, (2) patients were categorized based on the presence or absence of RVD, defined as the deviation of at least one measurement of RV function from the recommended normal range based on modality and the normal ranges established in each study, and (3) the Kaplan-Meier plot, along with their corresponding hazard ratios (HR), were available for the specified endpoints.

We applied the definition of HFpEF based on the specific criteria of each study. Of note, all studies defined HFpEF as the presence of HF signs and symptoms along with a left ventricular ejection fraction (LVEF) of 50 or greater, while some studies additionally included left ventricular diastolic dysfunction and NT-proBNP levels as part of the diagnostic criteria. The endpoint was defined as a composite of all-cause mortality, cardiac mortality, and HF hospitalization. Studies were excluded if they were reviews, meta-analyses, case reports or case series, animal studies, editorials, commentaries, abstracts, and studies not reporting the outcome of interest.

### Screening and selection of studies

The screening process was performed using the Rayyan web platform (Cambridge, Massachusetts, United States). After removing duplications, two authors (K.I. and R.NJ.) independently screened all studies. In cases of disagreement, a third reviewer (H.S.) provided expert opinion. The included studies were then imported into the EndNote software version 20 (London, United Kingdom). The study selection was also conducted independently by the same two authors (K.I. or K.B. and R.NJ.), with any conflicts resolved by a third reviewer (H.S.).

### Risk of bias assessment

The quality of the included studies was assessed by employing the ROBINS-E (Risk Of Bias In Non-randomized Studies - of Exposures) tool [20]. This tool is specifically designed for cohort studies and contains seven domains to be assessed in order to determine the quality of the study: bias due to confounding, bias arising from the measurement of the exposure, bias in the selection of participants into the studies (or into the analysis), bias due to post-exposure interventions, bias due to missing data, bias arising from the measurement of the outcome, bias in the selection of the reported results. Using this tool, two independent authors (K.B. and A.H.) evaluated the risk of bias in the included studies. Any conflicts were resolved by a third author’s opinion (H.S.). Based on the responses to the ROBINS-E questions, studies were categorized as having a low risk of bias, some concerns about the risk of bias, or a high risk of bias. A traffic light plot illustrating the quality assessment is provided in supplementary Fig. 1.

### Data extraction

Data extraction was performed independently by two authors (K.I. and R.NJ.). Disagreements between these two authors were resolved by a third author (H.S.). The following data was extracted from each study: the first author’s name, publication year, sample size, sex, mean age, heart rate, NYHA classification, body mass index, systolic blood pressure, diastolic blood pressure, glomerular filtration rate, creatinine, NT-proBNP or BNP, hemoglobin, total cholesterol, coronary artery disease, diabetes mellitus, hypertension, atrial fibrillation, smoking, chronic obstructive pulmonary disease, sleep apnea, pacemakers, cardiovascular disease, previous heart failure episode, hypercholesterolemia and drug history. The exposure was defined as right ventricular dysfunction, and the outcome was defined as a composite of all-cause mortality, cardiac mortality, and HF hospitalization in HFpEF. Data related to survival, including the Kaplan-Meier plot and HR, were extracted and used to reconstruct time-to-event data.

### Data synthesis and statistical analysis

We used the 2-stage approach as described by Liu et al. [21] based on the R package “IPDfromKM” (version 0.1.10). In the first stage, raw data coordinates (time and survival probability) were extracted from each Group (HFpEF patients with and without RVD) in each of the Kaplan-Meier curves. These curves were digitized using the Web Plot Digitizer tool for time-to-event outcomes. In the second stage, individual patient data were reconstructed by processing the extracted time points, survival probabilities, and the number of patients at risk. The reconstructed data from all studies were then merged to form a comprehensive dataset.

Hazard ratios (HRs) with 95% confidence intervals (CIs) were estimated using Cox proportional hazard models, fitted with a γ frailty term to account for study-specific random effects and unobserved heterogeneity. The proportional hazards assumption was evaluated using the Grambsch–Therneau test and Schoenfeld residual plots. When this assumption was violated, flexible parametric survival models with B-splines (Royston–Parmar models) were applied to model time-varying hazard ratios. A subgroup analysis was also conducted to evaluate the pooled prognostic effect of RVD based on studies that utilized echocardiographic parameters.

Meta-regression was performed to explore the potential impact of baseline covariates, including age, systolic pulmonary artery pressure (sPAP), the proportion of female patients, body mass index, glomerular filtration rate, the proportion of patients with coronary artery disease, the proportion of patients with diabetes mellitus, the proportion of patients with hypertension, and the proportion of patients with atrial fibrillation, on the effect sizes. This analysis helped to assess the extent to which variations in patient characteristics across studies could explain heterogeneity in the observed treatment effects. When Kaplan–Meier curves or further data were unavailable in the main manuscripts, principal investigators were contacted for clarification or to provide additional data.

The RMST was calculated as the area under the Kaplan-Meier survival curve for each group, with the difference in RMST (ΔRMST) providing an absolute measurement of effect size over time. Interaction effects between exposures and time were assessed using spline-based models, while landmark analyses were performed to focus on outcomes after the one-year time point during follow-up. All analyses were completed with R Statistical Software version 4.1.1 (Foundation for Statistical Computing).

## Results

### Systematic review

**Fig. 1:**
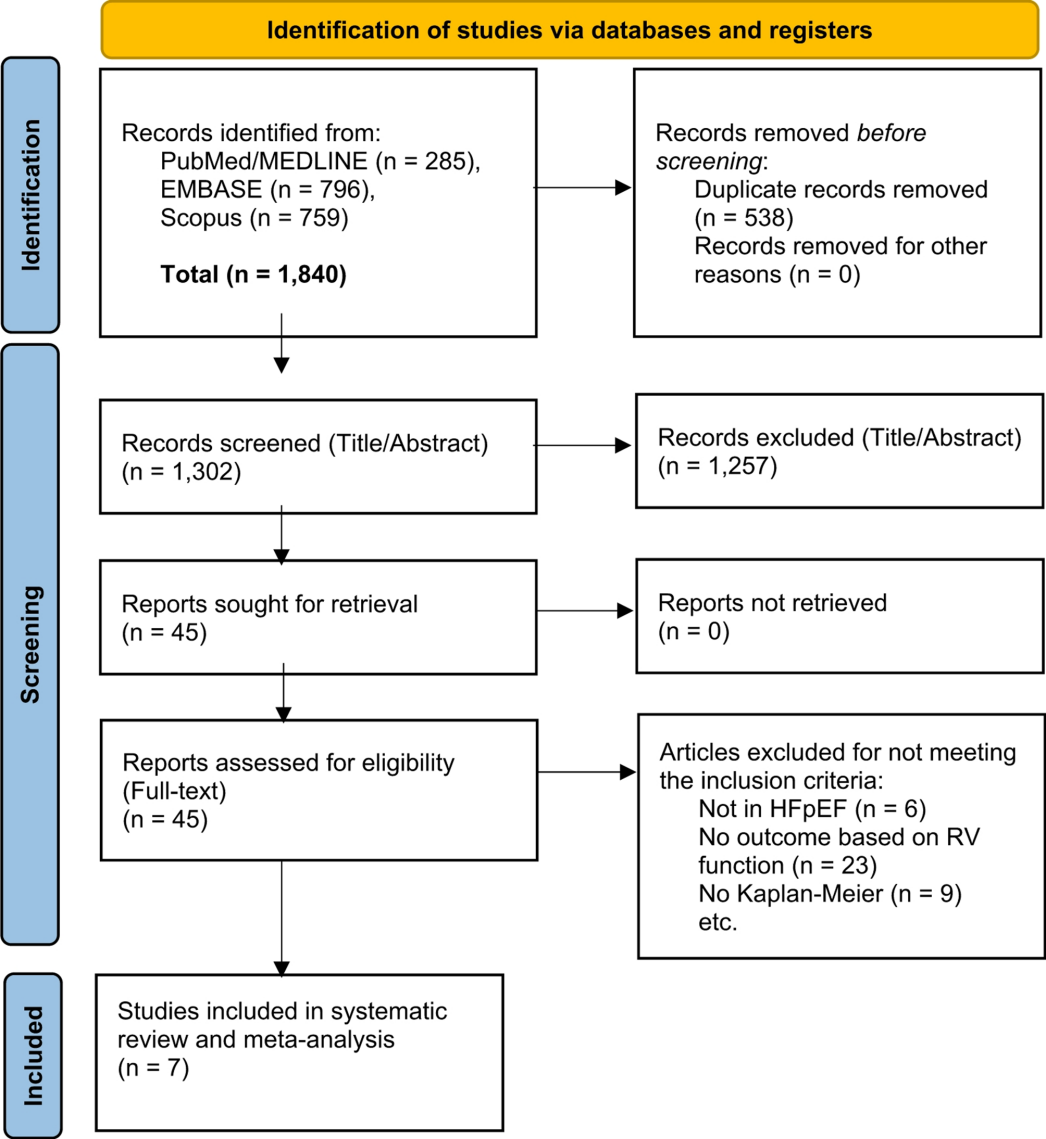
Flowchart of study selection

The systematic search identified 1,807 publications, from which seven studies met the inclusion criteria and were selected for meta-analysis, as outlined in Fig. 1. Table 1 provides a detailed summary of the included studies, which collectively encompassed 1936 individuals, of which 555 patients had RVD. Additionally, the quality assessment for each study is presented in the supplementary Fig. 1. The median follow-up period for included studies ranged from 17 to 55 months.

**Table 1:** Summary and baseline characteristics of included studies

Study ID	Study Design	Total population	Mean Age (SD)	Female (%)	RVD (%)	DM (%)	HTN (%)	AF (%)	RV assessment modality	RVD definition	Follow-up duration	Study endpoint	
Aschauer, 2016 [22]	Observational	171	70.0 (8.6)	110 (65)	33 (19)	69 (40)	161 (94)	97 (58)	CMR	RVEF *<* 45%	19.1 ± 12.9 months	combined endpoint of hospitalization for heart failure and/or death from cardiac causes	
Baruch, 2019 [24]	Observational	557	64.91 (20)	290 (52)	N/A	N/A	N/A	N/A	Echocardiography	TAPSE ≤ 15	36 months	All-cause mortality and cardiac mortality.	
Harada, 2019 [23]	Observational	322	76 (11)	176 (55)	N/A	65 (20)	281 (87)	74 (23)	Jugular pulse waveform with signal processing technique	Prominent ‘Y’ descent of the jugular venous waveform	33 ± 20 months	Sudden death, death from heart failure, or hospitalization for HFpEF	
Lejeune, 2020 [25]	Observational	149	78 (69)	91 (61)	28 (19)	61 (41)	138 (93)	86 (58)	Speckle-tracking echocardiographic	STE-RVGLS > -17.5%	30 ± 9 months	Composite of all-cause mortality or hospitalization for HF	
Melenovsky, 2014 [26]	Observational	96	71 (10)	57 (59)	32 (33)	46 (48)	90 (94)	41 (43)	Echocardiography	FAC < 35%	17.6 (4.8–35.5)	All-cause mortality	
Meng, 2021 [27]	Observational	81	61.7 (1.4)	28 (34)	N/A	36 (44)	46 (57)	10 (12)	Speckle-tracking echocardiographic	3D-RVFWLS < 22%	17 (11–36) months	death for HF or rehospitalization due to worsening of HF	
Mohammed, 2014 [28]	Observational	562	79 (5.6)	320 (57)	118 (21)	196 (35)	479 (85)	255 (45)	Echocardiography	TAPSE ≤ 15	55.2 months	Composite of all-cause mortality or hospitalization for HF	

One study reported RVD as RV Ejection Fraction (RVEF) *<* 45% using cardiac magnetic resonance imaging (CMR) [22]. Another study defined RVD as reduced distensibility of the right ventricle, as evidenced by the prominent ‘Y’ descent of the jugular venous waveform using signal-processing techniques [23]. The other five studies used different echocardiographic indexes to evaluate RV function. These indexes include tricuspid annular plane systolic excursion (TAPSE), speckle-tracking echocardiography-derived RV global or free wall longitudinal strain (RVGLS or RVFWLS), and RV Fractional Area Change (FAC).

### Meta-analysis

#### Prognostic impact of RV dysfunction

**Fig. 2:**
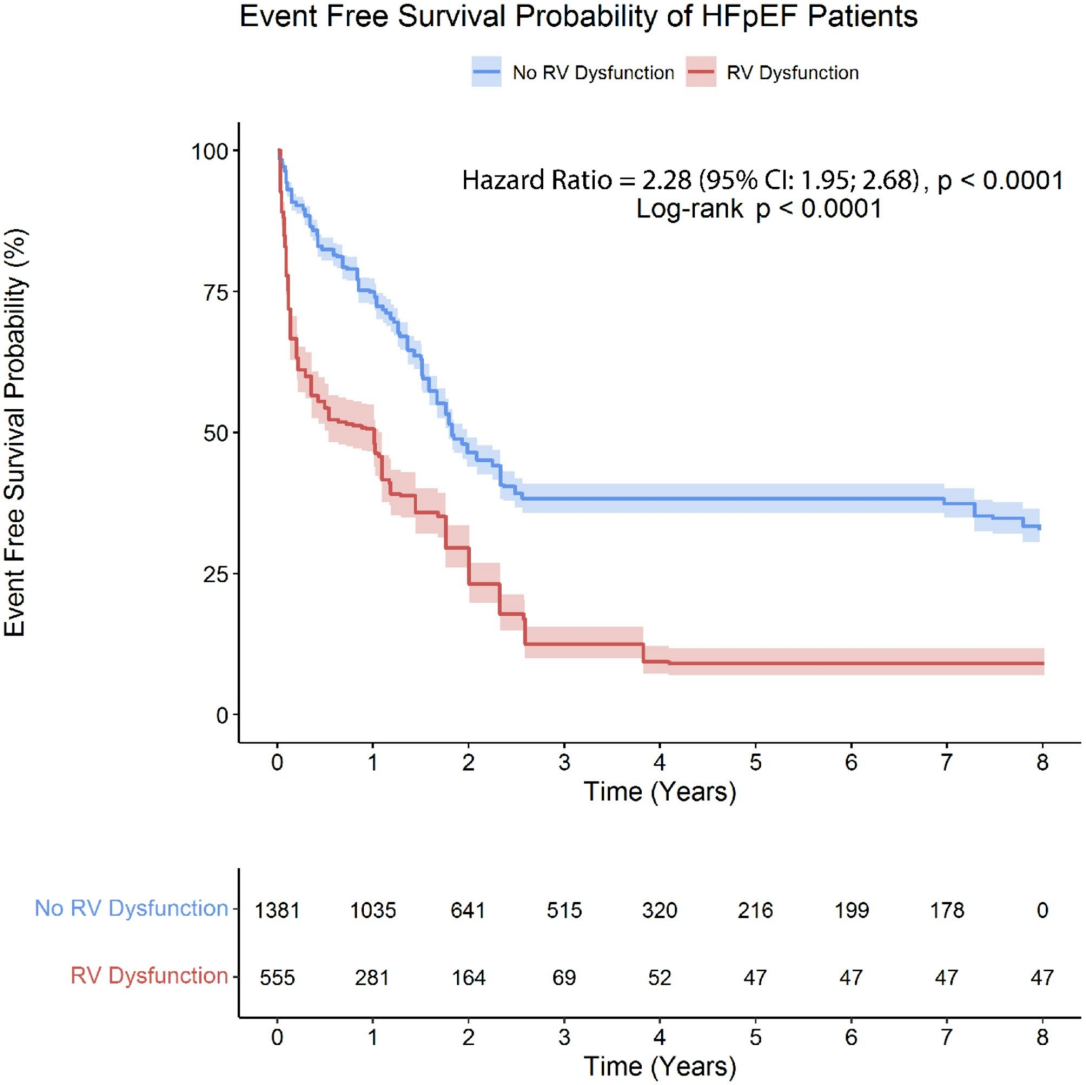
The Kaplan-Meier survival curves demonstrate a significant difference in event-free survival between HFpEF patients with and without RVD

The pooled prevalence of RVD among HFpEF was 41.2% (95% CI: 36.5; 45.9). As presented in Fig. 2, the Kaplan-Meier survival curves revealed a significant difference in event-free survival between HFpEF patients with and without RVD. Patients with RVD had a significantly higher risk of adverse outcomes compared to those without RVD, with an HR of 2.28 (95% CI, 1.95; 2.68, *p*-value < 0.001) in the eight-year follow-up after the diagnosis of RVD. The one-year landmark analysis revealed that during this period, the event-free survival probability for the RVD group declined sharply, with an HR of 2.32 (95% CI, 1.96; 2.72, *p*-value < 0.001) compared to those without RVD. Meanwhile, the HR for RVD in patients with HFpEF decreased to 1.92 (95% CI, 1.64; 2.24, *p*-value < 0.001) after the first year of follow-up. This indicates that the majority of the event-free survival disparity between patients with RVD and those without arises during the first year after an RVD diagnosis. (Supplementary Fig. 1).

**Fig. 3:**
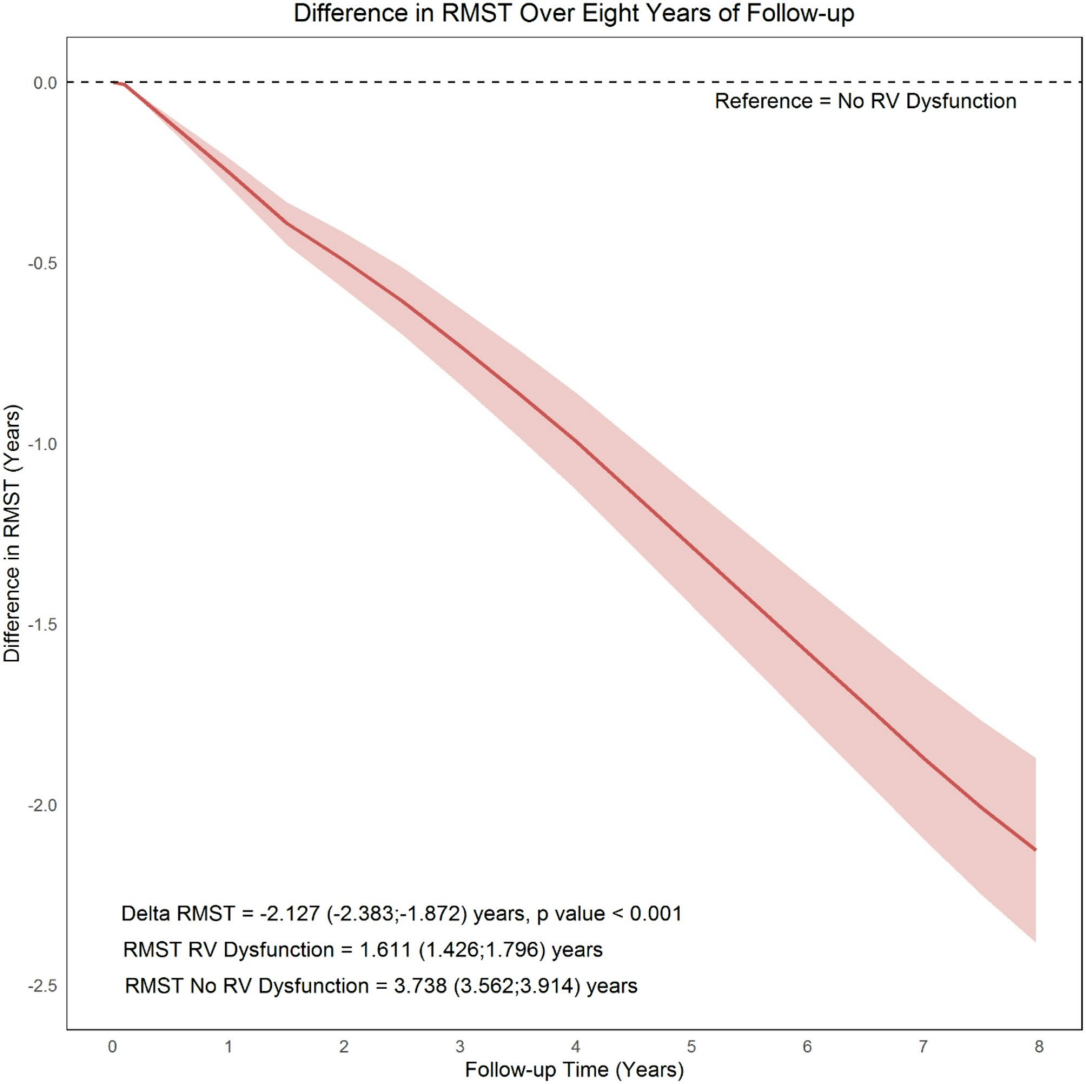
The trend of difference in Restricted Mean Survival Time over eight years of follow-up between patients with and without right ventricular dysfunction

Patients with RVD experienced a shorter RMST over eight years of follow-up. The RMST for patients without RVDs was 3.738 (95% CI, 3.562; 3.914) years, while for those with RVD, it was significantly lower at 1.611 (95% CI, 1.426; 1.796) years. The ΔRMST was − 2.127 (95% CI, -2.383; -1.872) years, indicating a reduced event-free survival rate in patients with RVD during the follow-up period (*p*-value < 0.001). (Fig. 3)

#### Subgroup analysis of prognostic impact of RV dysfunction

The subgroup analysis of five studies assessing RVD using echocardiographic parameters [24–28] showed similar trends to the primary analysis. Patients with RVD faced a considerably greater risk of adverse outcomes than those without RVD, with an HR of 2.17 (95% CI, 1.95; 2.53, *p* < 0.001) over the eight-year follow-up post-RVD diagnosis. The ΔRMST was − 2.007 years (95% CI, -2.300 to -1.714), indicating a reduced event-free survival rate comparable to primary analysis for patients with RVD during the follow-up period (*p*-value < 0.001). (Supplementary Fig. 3,4).

#### Meta-regression analysis

**Fig. 4:**
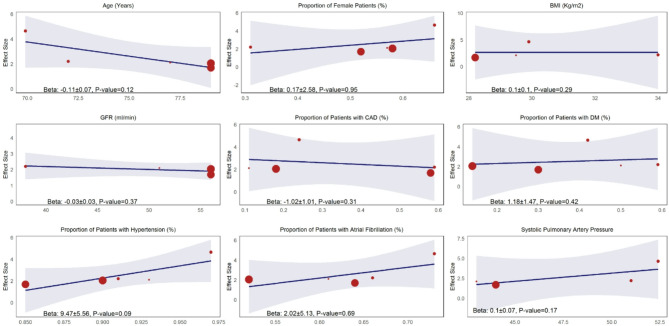
The meta-regression result for investigating the potential sources of heterogeneity in the prognostic impact of RVD in patients with HFpEF

Meta-regression analyses were performed to explore potential sources of heterogeneity across the included studies by examining key clinical and demographic characteristics (Fig. 4). The meta-regression results indicated that no single clinical or demographic variable significantly impacted the effect size of RVD, suggesting that a complex interplay of multiple factors likely influences the development of RVD in HFpEF.

## Discussion

### Overview of findings

In this meta-analysis, we examined the prognostic significance of RVD among patients with HFpEF by reconstructing individual time-to-event data via Kaplan-Meier curves. Our findings suggest that HFpEF patients with RVD experience significantly worse outcomes compared to those without RVD, with most of the disparity occurring in the first year following the RVD diagnosis. Furthermore, the RMST analysis indicated that RVD decreases event-free survival time by about 2.13 years during an 8-year follow-up.

### Pathophysiological insight into the RVD

The connection between pulmonary hypertension (PH) and HFpEF is well established, as resting PH is observed in at least two-thirds of these patients [9]. Similar to heart failure with reduced ejection fraction (HFrEF), elevated left-sided filling pressures lead to postcapillary PH, subsequently increasing strain on the RV and contributing to its dysfunction. Since the thin-walled RV operates against the pulmonary circulation, typically a low-pressure circuit, it is vulnerable to rises in afterload. In a subset of patients, a concurrent precapillary component may arise due to pulmonary venule muscularization, pulmonary capillary endothelial cell proliferation, and pulmonary arterial remodeling with intimal hypertrophy in response to volume overload [29]. The rise in RV pressure causes the interventricular septum to displace toward the left ventricle (LV), decreasing LV distensibility and preload and resulting in a low cardiac output state [30].

When the RV myocardium faces the increased afterload, it triggers the activation of various neurohormonal and molecular mechanisms. These include the release of cytokines, activation of the endothelin system, stimulation of the renin-angiotensin-aldosterone system (RAAS), engagement of the autonomic nervous system, and the release of natriuretic peptides [31]. To maintain adequate cardiac output, the myocardial wall of the RV becomes thicker. Eventually, this remodeling pattern causes a substantial mismatch between the oxygen demand and the myocardial blood supply of the RV. This leads to myocardial ischemia, oxidative stress, cytokine release, and, consequently, fibrosis and collagen production [32]. Increased catecholamine levels and excessive activation of β-adrenergic receptors in the context of RVD further promote maladaptive cardiac remodeling [33]. In addition to the cellular and molecular processes responding to elevated afterload, alterations in the pulmonary vasculature also contribute to compromised pulmonary function and gas exchange [29].

On the other hand, a number of these pathways may also be triggered in HFpEF, even if PH is not present. This could be because of the direct consequences of co-morbid conditions like obesity, diabetes mellitus, and hypertension, which trigger the release of reactive oxygen species through inflammatory cytokines, cause endothelial dysfunction, and negatively remodel cardiomyocytes [34].

### RVD assessment challenges

In this meta-analysis, we pooled data from studies that utilized various echocardiographic and MRI parameters, as well as one study that employed a waveform signal processing technique to define systolic RVD. We could not standardize RVD definitions across studies, as each employed distinct parameters to define systolic RVD.

The RV systolic function arises from its longitudinal and radial contractions. There is still a lack of normative data concerning the contributions of longitudinal, radial, and anteroposterior components of RV wall motion to overall ejection. While earlier research indicated that longitudinal shortening primarily drives RV pump function under physiological conditions [35], more recent studies point to a comparable significance of both longitudinal and radial motions [36, 37]. However, under conditions with pressure overload, such as HFpEF, the circumferential fibers in the RV wall tend to dominate [38]. Studies frequently report a significant reduction in radial contraction, indicating that radial contraction is a more reliable predictor of RV pump function and screening criterion for pulmonary hypertension in HFpEF than longitudinal shortening [7, 39].

The assessment of RV function is typically based on TAPSE, which only accounts for the longitudinal shortening of the RV, similar to RVGLS and RVFWLS [40]. On the other hand, FAC accounts for both the longitudinal shortening and radial thickening of the right ventricular free wall. Moreover, FAC also considers the contribution of the interventricular septum when evaluating right ventricular systolic function [41]. This metric proves particularly valuable for assessing RV systolic function during the early postoperative phase or is affected by loading conditions and geometric changes seen in conditions like HFpEF when TAPSE may be underreported [42]. Additionally, FAC strongly correlates with RVEF obtained from CMR, making it a key measure of both longitudinal and radial RV functions [43].

The inconsistency in these diagnostic criteria results in notable variability among the involved studies, potentially affecting the comparability of their results. Variations in diagnostic methods may impact the classification and severity of RVD, subsequently influencing the prognostic outcomes observed in various studies. Furthermore, the latest position statement from the Heart Failure Association of the European Society of Cardiology suggests that to consider RVD in HFpEF, at least two echocardiographic parameters for RV systolic function should fall below the recommended cut-off, or an RV ejection fraction measured with CMR should be below 45% [7]. A recent study also demonstrated that having two or more abnormal RV function parameters was associated with a significantly worse prognosis [44]. However, the studies we included assessed RVD solely based on a change in one parameter of RV systolic function.

Nevertheless, while acknowledging these variabilities and challenges, the outcomes of our pooled analysis should be regarded as significant as they provide new insights regarding the magnitude of the effect of RVD in patients with HFpEF, although they necessitate careful interpretation. Furthermore, it is important to note that the pooled prognostic results of RVD, defined in various ways, have been previously validated and published [13].

### Prognostic significance of RVD in HFpEF

The RV has an important role in maintaining circulation, especially in cases of left-sided HF and PH [45, 46]. A number of studies have evaluated the prognostic significance of RV function in patients with HFpEF. It has been shown that RVD is associated with increased all-cause and cardiovascular mortality and hospitalizations related to heart failure [47]. Melenovsky et al. indicated a 2.2-fold elevated risk of all-cause death for each 7% reduction in FAC after adjustments for pulmonary pressures [26]. In another investigation Using CMR, the hazard ratio for the outcome was 4.9 for HFpEF patients with an RVEF below 45%, showing a significantly increased risk for a combined endpoint consisting of hospitalization for heart failure and/or death from cardiac causes [22]. Furthermore, pooled data from a meta-analysis showed that the mortality risk escalates by about 26% for every 5 mm reduction in TAPSE and by approximately 16% for every 5% decline in FAC [48].

The assessment of RV function and its prognostic significance has been extended beyond conventional imaging techniques. In recent years, CMR has become the preferred modality for evaluating right ventricular anatomy and function. Due to its excellent spatial and temporal resolution, together with high reproducibility, CMR-derived volumes and ejection fraction are widely regarded as the gold standard for assessing right ventricular function and volume [49]. Furthermore, Lejeune et al. found that STE-derived RVGLS serves as a better prognostic marker than traditional measures such as TAPSE or FAC. Specifically, patients with impaired RVGLS (> -17.5%) experienced significantly lower survival rates, indicating that strain imaging should become a standard part of evaluating HFpEF [25]. In a similar vein, Meng et al. emphasized the benefits of 3D-STE for assessing RV volumes and strain [50]. This technique provides a more thorough evaluation compared to 2D methods by overcoming challenges like geometric modeling limitations and foreshortening effects, thereby offering more accurate insights into RV mechanics. The assessment of RV distensibility as an indicator of RV function has also been investigated. This technique identified a prominent “Y” descent jugular venous waveform as a strong indicator of poor prognosis in HFpEF, offering a straightforward yet effective tool for patient stratification [23].

All of these various modalities have shown a notable increase in mortality and cardiovascular events in HFpEF patients associated with RVD. Our findings are consistent with previous studies, indicating that patients with RVD are at a greater risk of experiencing adverse clinical outcomes within the first year of diagnosis compared to HFpEF patients without RVD. This risk also remains higher in an eight-year follow-up course. In addition, according to our RMST analysis, RVD is associated with a mean of 2.13-year decrease in event-free survival.

The observed survival trend can be attributed to the complex interaction between RVD and systemic factors. RVD worsens systemic venous congestion and lowers cardiac output, both of which are key factors contributing to mortality [51]. It’s important to note that patients with RV dysfunction often deal with more advanced diseases. They typically experience more severe symptoms and have additional health issues [52]. These patients are more likely to suffer from atrial fibrillation, have pacemakers, have coronary artery disease, and be on long-term diuretic therapy, which points to a later stage of HFpEF. In addition, male gender has been shown to be a predicting factor for RVD development [26, 28].

Of note, all included studies found that RVD is independently associated with poorer outcomes in HFpEF patients, even after multivariate Cox hazard analysis was adjusted for confounders, specifically for pulmonary artery pressure. In our study, we assumed that all published Kaplan-Meier curves were designed based on the models adjusted for most covariates in each study, although only one of the included studies clearly stated that the Kaplan-Meier curve was adjusted for covariates [25]. As a result, it can be concluded that our reconstructed individual patient data have also been adjusted for confounders. However, it should be acknowledged that studies have adjusted the results for different covariates, which can be another source of heterogeneity between the studies. On the other hand, since the established method used in this study only reconstructs patients’ data regarding whether the patient experienced an event and when the event happened, it cannot apply baseline characteristics to each reconstructed patient. As a result, independently adjusting the hazard ratio for confounders after pooling the extracted data is not feasible in this method. Finally, considering that each study found significant results after adjusting for confounders, we can cautiously conclude that the pooled result should remain significant, as our results showed, and RVD may be independently associated with poorer outcomes in patients with HFpEF. However, the observational nature of these studies limits our ability to infer causality regarding RVD’s direct contribution to poorer outcomes as an independent risk factor versus its role as merely an indicator of advanced disease.

### Clinical implications

#### Early and accurate diagnosis of RVD

RVD should be routinely evaluated in clinical practice to recognize high-risk HFpEF patients according to its additive prognostic value. Echocardiography is generally the first-line modality in assessing RV function. TAPSE is one of the most often utilized echocardiographic parameters in this term as an independent prognostic metric [53, 54]. Although previous investigations commonly used a cut-off of < 16 mm, the current suggested lower limit cut-off for TAPSE is < 17 mm [55]. FAC is also frequently employed with a lower limit of normal < 35% [55]. Since the greatest data about prognosis is available for TAPSE and FAC, it is preferable to evaluate these two markers in all HFpEF patients [26, 56]. RV longitudinal strain and RV S’ are additional echocardiographic indicators of RVD [57–59]. RV longitudinal strain is advantageous due to its angle independence and its capacity to identify minor, localized myocardial alterations in HFpEF [59]. Additionally, since the LV has tethering effects because both ventricles share myocardial muscle fibers, TAPSE and RV S’ may sometimes be mistakenly raised when the LV is hyperdynamic. These tethering effects may have less of an impact on right ventricular strain in this situation [31].

Clinicians have, up to recent years, depended on echocardiography to evaluate RV function. However, echocardiography might have some limitations, especially in patients with HFpEF who also typically have obesity or COPD. The RV’s intricate anatomy makes it difficult to measure RVEF with the same precision and repeatability as LVEF. The accuracy of RVEF determination is further influenced by a number of factors, including load dependency, complex geometry, and retrosternal position [60].

Cardiovascular magnetic resonance imaging (MRI), therefore, has become the preferred method in this context due to its high repeatability and great spatial and temporal resolution. The CMR-derived volumes and RVEF measurements are becoming more widely recognized as the gold standard. The most often used cut-off for RVD is an EF < 45%, which is linked to a poorer prognosis in HFpEF [22]. CMR also makes it possible to quantify blood flow and characterize tissue, which might provide further diagnostic hints about the origin of RVD. For instance, T1 mapping or late gadolinium enhancement can be used to diagnose myocardial fibrosis [61].

#### The available treatment options

The pronounced and rapid drop in RMST, in addition to high hazard ratios, particularly during the first year of RVD diagnosis, underscores the need for prompt treatment for HFpEF patients with RVD. These patients might benefit from more proactive management strategies, including starting guideline-recommended treatments sooner and having more frequent follow-ups.

The primary goal of treatment should be to reduce left ventricular filling pressure. This will help to (1) modulate the abnormal pulsatile loading on the RV and (2) prevent fluid swelling from pulmonary capillaries to the interstitium, which will raise transmural arteriolar pressure and cause pulmonary arterial compliance loss [62]. Therapeutic approaches should specifically focus on contrasting the various pathways that contribute to lung capillary stress failure and remodeling [63].

Phosphodiesterase inhibitors (PDIs) are a class of medications used in the management of PH, with their effects believed to arise from selective vasodilation of the pulmonary circulation [64]. Although sildenafil, a PDI, has been linked to improvements in pulmonary pressures and RV function, the Phosphodiesterase-5 Inhibition to Improve Clinical Status and Exercise Capacity in Heart Failure with Preserved Ejection Fraction (RELAX) trial showed that, when compared to a placebo, it had no effect on clinical status or exercise capacity in HFpEF with or without PH [65].

In a translational porcine model of postcapillary chronic PH, β3AR (beta-3 adrenergic receptor) agonists have recently been demonstrated to enhance pulmonary hemodynamics, RV remodeling, and pulmonary vascular proliferation [66]. According to advanced cardiac imaging, the SPHERE-HF trial, the first clinical study to evaluate the potential effect of β3 adrenergic agonists in PH, mirabegron significantly improved the right ventricular ejection fraction. However, the functional class and quality of life showed no improvement in this study [67]. Interatrial communication devices are also innovative nonpharmacological methods for treating HFpEF. This device requires the absence of pulmonary hypertension at rest and adequate right ventricular function for implantation eligibility. The potential of this strategy to postpone or avoid the development of RV dysfunction also warrants further exploration [68].

Additionally, since AF, coronary artery disease, excess body weight, and aberrant cardiac hemodynamics are possible modifiable risk factors associated with the development of RVD, clinical studies focusing on treating these risk factors are recommended to ascertain if RV structure and function may be preserved [69]. Given how RVD affects prognosis, targeting RV-specific issues could offer new treatment opportunities. Future studies should investigate whether early and aggressive treatment of RVD and its complications can improve the prognosis of HFpEF patients.

### Strengths and limitations

Despite the high quality of previous meta-analyses, the majority of them pool their data mainly employing random effects models to provide incidence rate ratios, odds ratios (OR), or risk ratios (RR) as final metrics. Some investigators combine median survival times, incidence rate ratios, or event rates derived from survival estimates at certain time intervals, or directly assess HRs across the investigations. All these methods have been shown to be constrained and inadequate since they fail to allow the construction of pooled Kaplan-Meier curves and neglect fundamental principles of survival analysis, including censoring and the proportional hazards assumption [70].

To address the inconsistent findings arising from these divergent methodologies, the “curve approach” has become the prevailing norm for the meta-analysis of aggregated time-to-event data. This method reconstructs individual patient data using published Kaplan-Meier plots from the included studies [21]. Our meta-analysis employs a robust method, including the reconstruction of Kaplan-Meier survival data from original studies, to assess how RVD impacts outcomes in patients with HFpEF. By reconstructing this curve, we were able to add time-to-event data to our analysis to evaluate the prognostic significance of RVD more comprehensively. RMST analysis provided a way to measure how RVD impacts survival time, showing the real-world effects beyond traditional hazard ratios.

However, there are some limitations to consider. While we gathered a large sample size by combining data from several cohorts, this meta-analysis mainly includes retrospective observational studies. Furthermore, the Kaplan-Meier-derived time-to-event data were not based on pooling original datasets but depended on the quality and accuracy of the published curves, which might introduce some inaccuracies. The variations in assessing RV function across the studies included may also have influenced the results. We could not entirely dismiss the possibility of residual confounding, even after adjusting for key factors.

The diversity in diagnostics presents a significant challenge for evaluating the prognostic impact of RVD. Although these imaging methods are frequently used in clinical settings, the lack of a universally recognized “gold standard” for RVD definition complicates the generalization of our findings, potentially leading to some levels of inaccuracy in conclusions. Establishing standardized diagnostic criteria for RVD across various studies would improve the consistency and comparability of future research. Considering these factors, we recommend that future studies focus on a standardized definition of RVD, particularly emphasizing the development of consensus guidelines that enable a more uniform and precise evaluation of right ventricular function and its prognostic effect in both clinical and research contexts.

## Conclusion

This meta-analysis underscores that the development of RVD in HFpEF is linked to significantly increased composite outcomes of all-cause death, cardiac death, and HF hospitalization and shorter event-free survival. The risk of developing adverse outcomes is more evident in the first year of RVD diagnosis, emphasizing the importance of prompt detection and treatment of this condition.

## Electronic supplementary material

Below is the link to the electronic supplementary material.

Supplementary Material 1

## Data Availability

No datasets were generated or analysed during the current study.
