# Supplementary material for: Prognostic significance of right ventricular dysfunction in heart failure with preserved ejection fraction: a meta-analysis of reconstructed time-to-event data
Source: Echo Res Pract. 2025 May 29;12:13. doi: 10.1186/s44156-025-00080-5 (PMC12121155; doi:10.1186/s44156-025-00080-5)
Supplement: Supplementary file 1 — Supplementary Material 1 [file 44156_2025_80_MOESM1_ESM.docx]

Supplementary figures


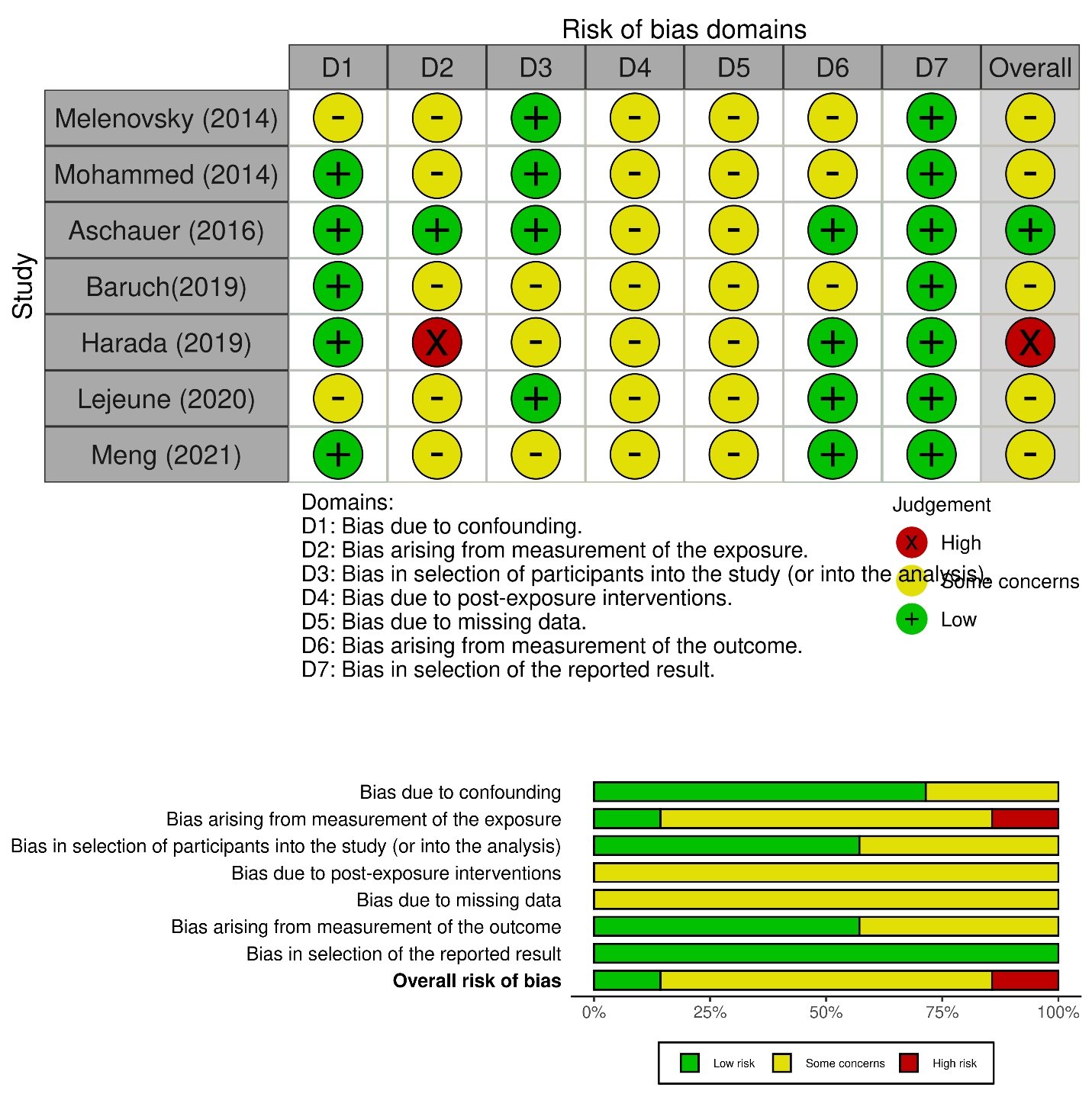


Supplementary Figure 1- The summary of risk of bias assessment using the ROBINS-E tool


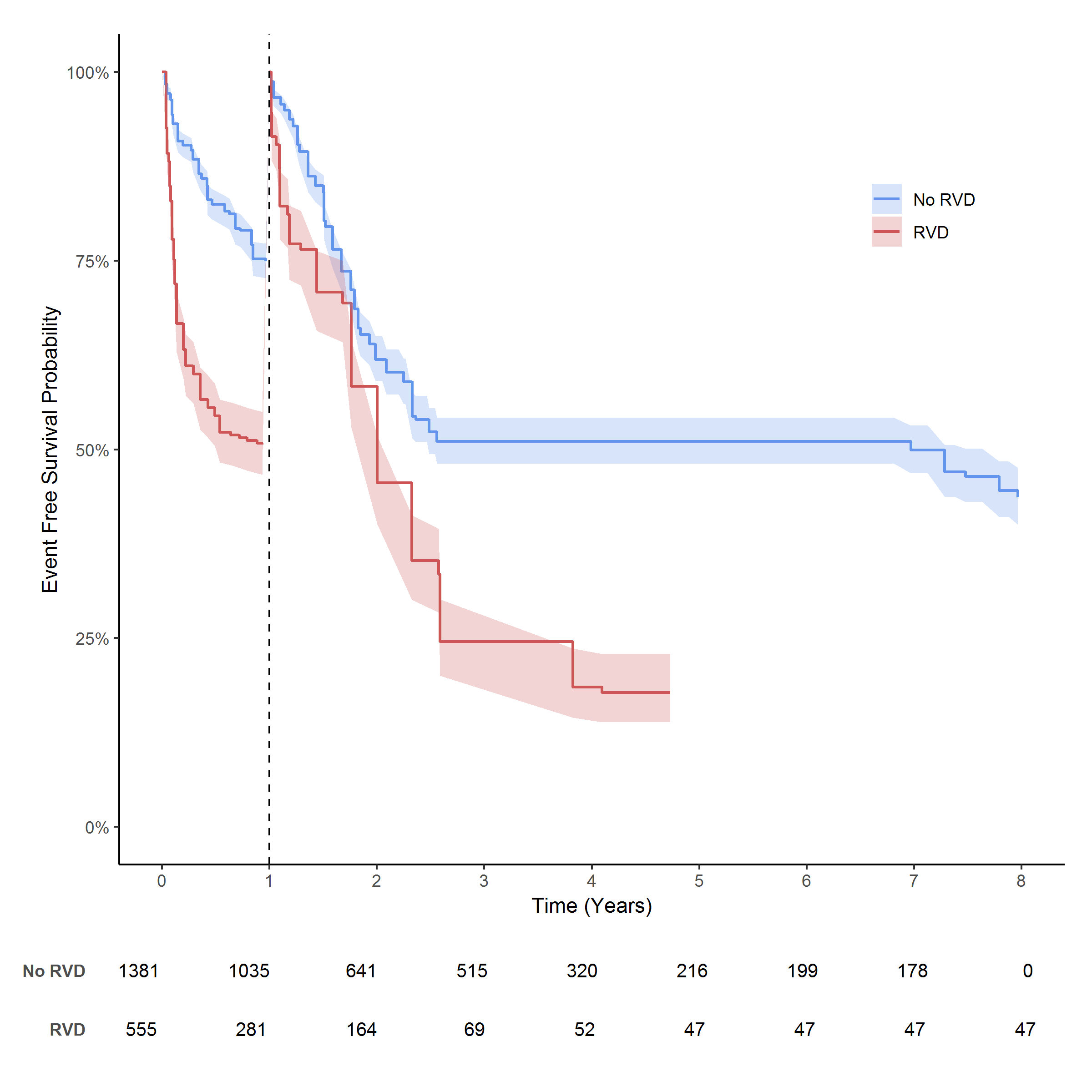


Supplementary Figure 2- One-year landmark analysis. In the first year of follow-up, the event-free survival in patients with RVD dropped more sharply compared to patients without RVD.


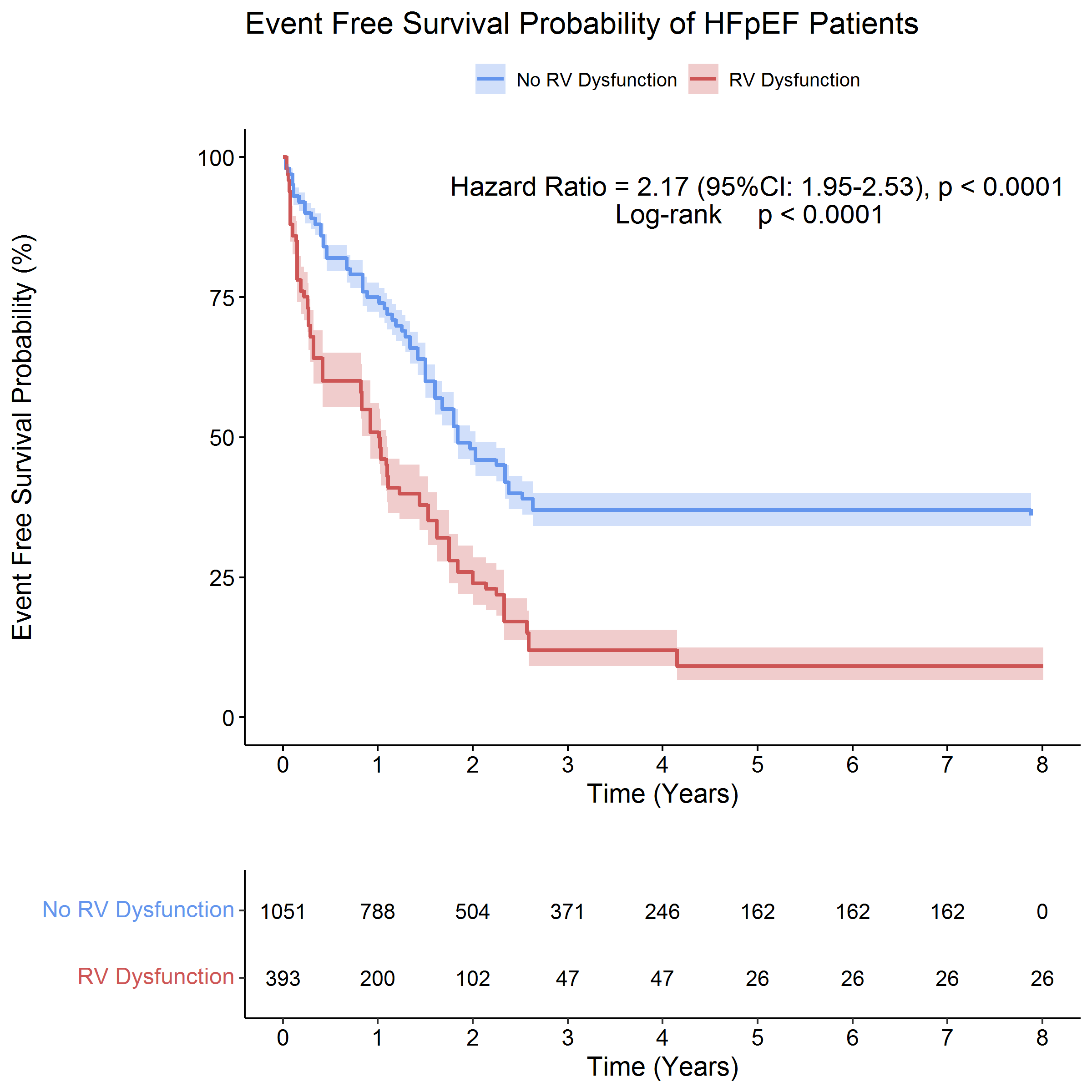


Supplementary Figure 3- Subgroup analysis on event-free survival in studies defined RVD with echocardiographic parameters. The results show a significant difference between patients with and without RVD.


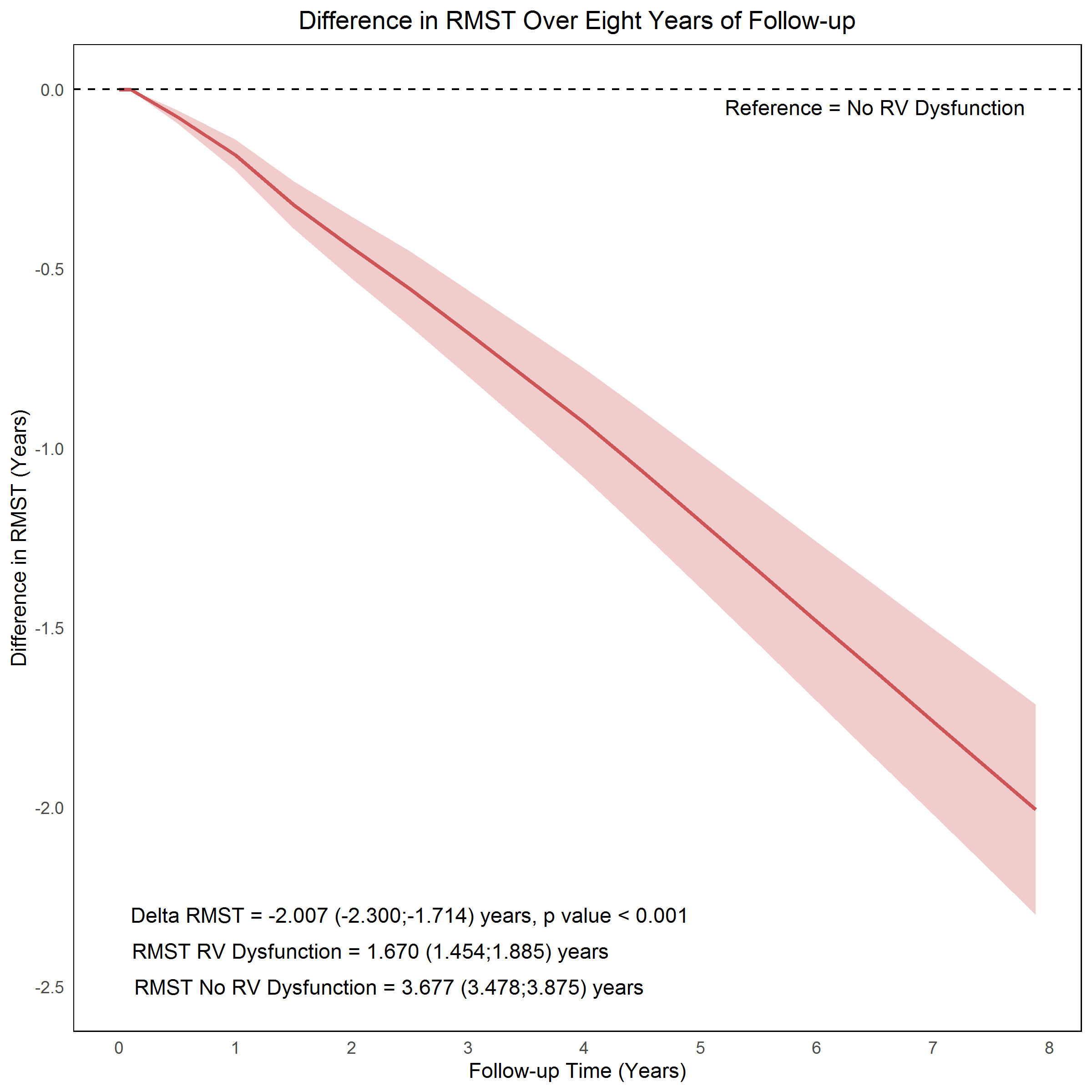


Supplementary Figure 4- The difference trend in Restricted Mean Survival Time over eight years of follow-up between patients with and without right ventricular dysfunction in subgroup patients with an echocardiographic definition of RVD.
